# A Topology Control with Energy Balance in Underwater Wireless Sensor Networks for IoT-Based Application

**DOI:** 10.3390/s18072306

**Published:** 2018-07-16

**Authors:** Zhen Hong, Xiaoman Pan, Ping Chen, Xianchuang Su, Ning Wang, Wenqi Lu

**Affiliations:** 1 Faculty of Mechanical Engineering & Automation, Zhejiang Sci-Tech University, Hangzhou 310018, China; xmanpan055@gmail.com (X.P.); luwenqi@zstu.edu.cn (W.L.); 2 School of Electrical & Computer Engineering, Georgia Institute of Technology, Atlanta, GA 30332, USA; 3 Zhejiang Provincial Testing Institute of Electronic Information Products, Hangzhou 310007, China; 4 School of Information Science & Technology, Zhejiang Sci-Tech University, Hangzhou 310018, China; xianchuangsu@gmail.com; 5 School of Public Administration, Zhejiang University of Finance & Economics, Hangzhou 310018, China; nwang@zufe.edu.cn

**Keywords:** topology control, energy-efficiency, energy balance, underwater wireless sensor networks, acoustic communication, shallow water, IoT

## Abstract

As part of the IoT-based application, underwater wireless sensor networks (UWSN), which are typically self-organized heterogeneous wireless network, are one of the research hot-spots using various sensors in marine exploration and water environment monitoring application fields, recently. Due to the serious attenuation of radio in water, acoustic or hybrid communication is a usual way for transmitting information among nodes, which dissipates much more energy to prevent the network failure and guarantee the quality of service (QoS). To address this issue, a topology control with energy balance, namely TCEB, is proposed for UWSN to overcome time-delay and other interference, as well as make the entire network load balance. With the given underwater network model and its specialized energy consumption model, we introduce the non-cooperative-game-based scheme to select the nodes with better performance as the cluster-heads. Afterwards, the intra-cluster and inter-cluster topology construction are, respectively, to form the effective communication links of the intra-cluster and inter-cluster, which aim to build energy-efficient topology to reduce energy consumption. With the demonstration of the simulation, the results show the proposed TCEB has better performance on energy-efficiency and throughput than three other representative algorithms in complex underwater environments.

## 1. Introduction

The Internet of Things (IoT) is a widely spread information technology using smart sensors, RFID, smartphones and various communication protocols [1]. In recent years, there are many IoT-based application scenarios, such as smart cities and smart environments (e.g., smart home), environmental monitoring and disaster prevention, intelligent transportation and auxiliary navigation as well as battlefield surveillance. As an important part of IoT technologies, underwater wireless sensor networks (UWSN) are typically a self-organized heterogeneous wireless network, which is composed of many multi-functional underwater micro-sensor nodes with acoustic communication links [2]. With different assembled sensors, nodes are used to collaboratively sense the underwater environment and collect data to perform on-line real-time monitoring tasks. Specially, each node can be controlled to float up or down by its buoy and pumping to freely anchor a certain region to form a two-dimensional or three-dimensional network. The collected information is forwarded on the acoustic modem after data processing and storage. UWSN can be applied to marine exploration, aquaculture, water quality monitoring and pollution prevention [3,4].

Since radio is seriously attenuated in water, acoustic communication is almost the only effective way for underwater wireless transmission. Nevertheless, compared with the traditional radio, underwater acoustic communication is greatly affected by poor conditions, such as absorption, scattering, multipath interference and Doppler effect. Usually, the speed of acoustic transmission is 1500 m/s underwater [3] while it spreads almost 3 × 10${}^{8}$ m/s in the air by the radio. Obviously, there are five orders of magnitude difference between underwater and air communication. Due to the low rate propagation speed, the transmission delay of the underwater acoustic channel is always much higher in UWSN than the terrestrial wireless sensor networks (TWSN) [5]. This not only results in less throughput and lower communication efficiency, but also leads to increasing the probability of communication collision among the nodes. On the other hand, the reliability of data transmission is reduced by the multipath effect of signal propagation. It needs to resend data multiple times, which means it costs several time more energy consumption. In addition, a part of nodes may be unavailable to work during the network running, e.g., sudden failure or energy exhaustion, which cannot guarantee the connectivity and coverage of the entire network.

To solve above issues, an effective topology control scheme for UWSN is particularly important. Generally, topology control which mainly consists of topology construction and topology maintenance is one of the efficient solutions for saving energy and prolonging lifetime [6]. Thus, the main purpose of topology control is to close the idle nodes or cut off the unnecessary links. Basically, energy-efficiency is the most important target for UWSN to reach. This is all because the energy is dissipated much more on underwater acoustic or hybrid communication. Furthermore, the quality of service (QoS) such as packets throughput should be guaranteed during the communication procedure, while preserving the connectivity and coverage of the entire network.

This paper focuses on energy-efficient-based topology control for UWSN with clustering strategy using tree approach. We propose TCEB, a topology control algorithm with energy balance, which addresses the problem of how to find a reasonably reduced topology and the packets forwarding route. In TCEB, the whole process is mainly divided into three aspects: the non-cooperative-game-based cluster-head selection, the intra-cluster topology construction and the inter-cluster topology construction. Each part of work is performed to ensure energy-efficiency and make the whole underwater network load balance. Consequently, with energy conservation as the target, the main contributions of this paper are summarized as follows. (1) The underwater network model and its energy consumption model with proprietary features in the shallow water are considered as the basis. Simultaneously, non-cooperative game theory is introduced to the cluster-head selection, which can acquire the relatively optimal set of the cluster-heads and ensure the energy consumption of the whole network balance as much as possible. (2) Both intra-cluster and the inter-cluster topology construction are key sub-processes to find the optimum relay nodes to perform the forwarding task. It would help to reduce some part of nodes burden and make the network higher energy-efficiency.

The rest of the paper is organized as follows. In Section 2, we describe the related work on topology control techniques for UWSN. The system models which include network model and energy consumption model of UWSN are presented in Section 3. Moreover, in Section 4, the algorithm TCEB, which covers cluster-head selection, intra-cluster topology construction and inter-cluster topology construction, is proposed and analyzed in detail. The performance of TCEB is evaluated in Section 5. Finally, we conclude this paper in Section 6.

## 2. Related Work

Due to the characteristics of the underwater acoustic channel, e.g., high propagation delay, high bit error rate, multi-path effect and Doppler effect, there is a larger difference between the UWSN and TWSN. In UWSN, we must take more care about the complex underwater environment, especially for the contaminated water. It needs an extra design for real scenarios and computational complexity. Consequently, the commonly traditional topology control in the TWSN cannot be directly applied to the UWSN. We need to redesign a series of algorithms or protocols to meet the requirements of UWSN according to the underwater environment conditions.

Energy-efficiency is one of the most important targets to design a topology control mechanism in UWSN. To address this aim, clustering technology is the most common and available scheme for reliable communication and energy conservation. LEACH [7], which is a typical cluster-based topology control algorithm, periodically selects cluster-heads and uniformly drains energy by role rotation with data fusion strategy. Nevertheless, it is more suitable for ideal network model in the homogeneous TWSN, and there are some problems in its own clustering mechanism, such as uneven distribution of cluster-heads and poor energy balance. EDCS [8] is another cluster-based scheme which is proposed for heterogeneous network scenarios. Compared with the LEACH, EDCS achieves the better energy-saving effects for more general heterogeneous network because it introduces more accurate average network estimation and gravitational strategies. However, this does not apply to UWSN, which is a more complex and general heterogeneous network.

Recently, many topology control algorithms have been proposed for UWSN applications [9]. As one of the most important factors, energy-efficient topology control is firstly focused on by scholars under UWSN. Coutinho et al. [10] proposed two mechanisms, namely, the centralized topology control and the distributed topology control, to organize the network by some nodes depth adjustment. Combined with the corresponding geographic forwarding protocol, the data packets delivery ratio can be achieved into a higher level even in such hard scenarios (e.g., very sparse or dense network), while the energy consumption can also be reduced. Nevertheless, it must have the precise node position information which is provided by the extra localization system; simultaneously, the network cannot guarantee connectivity because it may have isolated nodes. Jouhari et al. [11] proposed a new kind of greedy forwarding (NGF) strategy for the geographic-based topology control in UWSN using the splitting mechanism with Chinese remainder theorem. In NGF, it is effective for more than two nodes to participate in the forwarding of one packet instead of selecting only one node as the next-hop, because the source node can reduce the number of bits transmission by splitting mechanism. NGF can increase the lifetime and other network performance, but it needs an extra strategy to solve isolated and void nodes problem appearing in the topology formation result.

A scale-free network model for calculating the edge probability is used to randomly generate the initial topology in [12]. Subsequently, a complex network theory-based topology control strategy is put forward to build a dual-clustering structure with two kinds of cluster-heads to ensure the connectivity and coverage, as well as optimize network energy consumption and propagation delay. It indicates the scale-free model can be applied in UWSN hierarchical topology but it may not satisfy the demand of specific applications. Specially, they do not mention the tradeoff between cluster-heads vulnerability and the cost from role rotations when using clustering technology. Further, to meet the requirement of diverse coverage in UWSN, Liu et al. [13] proposed the traversal algorithm for diverse coverage (TADC) and the radius increment algorithm for diverse coverage (RIADC), respectively. Actually, both TADC and RIADC satisfy the coverage for topology control through altering the sensing radii of nodes. However, the only difference is TADC only adjusts the sensing radius of one node at each round while multiple nodes in RIADC may increase their sensing radii in each round simultaneously.

Because to UWSN is typically an opportunistic network, QoS-based target is a focus issue that we are always concerned about. As we know, the links for the instant message in UWSN are always unstable and cut off [14,15]. The probabilistic multipath routing behavior driven by the opportunistic routing protocols is modeled in [15]; simultaneously, the probabilistic-based multipath centrality metric is proposed to measure the importance of UWSN to the data delivery task. It can be used to guide topology control to make better network performance because of identifying the critical nodes. In [16], to gain the preferable throughput efficiency of the network, the improved distributed topology control (iDTC) and the power adjustment distributed topology control (PADTC) are, respectively, proposed to guarantee the delivery of data by dealing with communication void problem of geographic opportunistic routing. With the depth adjustment and power adjustment of a void node, both protocols can obtain better energy efficiency and perform minimum displacement in the case of a void node while maintaining the same throughput.

On the other hand, due to the characteristic of water flowing, node mobility is another critical factor in UWSN [17]. Therefore, many kinds studies discuss mobility-based topology control techniques. Zhang et al. [18] and Liu et al. [19] proposed either mobility models for specialized applications or mobility-targeted topology control algorithms for movable UWSN. In addition, topology control can also contribute to other aspects of UWSN, e.g., localization technique. Usually, the unlocalized node can find its location by utilizing the spatiotemporal relation with the reference nodes; however, most nodes lack the required number of the reference nodes in the sparse scenario. To address this problem, Misra et al. [20] proposed an opportunistic localization by topology control (namely, OLTC) for sparse UWSN. In OLTC, some reference nodes are discovered through the topology construction process, while a game-theoretic model based on single-leader–multi-follower Stackelberg game for topology control is established to describe the relationship between the unlocalized and the localized nodes. Consequently, with the help of OLTC scheme, the localization coverage and the energy-efficiency can be promoted better than before.

In summary, topology control is indeed one of the worthy techniques for UWSN to study, even if research scholars focus on the different points and targets. From almost all literature, the energy-efficiency is still the common point which is only concerned about for the whole network. However, the current study for topology control in UWSN has the following drawbacks. (1) The overly idealized network model: The current assumed network model should be application-oriented built for the real environment. (2) Topology control scheme without preferable load-balance: Since energy is the key point for the underwater IoT-based application, topology control without preferable load-balance cannot ensure energy conservation and limits the further application. Consequently, we propose a topology control algorithm with energy balance (namely, TCEB) for UWSN, which contributes to a better energy-efficiency under such complicated underwater communication mode. The main target is to make underwater network load-balance and consume energy efficiently so that the lifetime of the network would be prolonged.

## 3. System Model

### 3.1. Network Model

Actually, the underwater network model and its corresponding communication are not the same in shallow water and deep water. In this paper, we only focus on the shallow water environments since most IoT-based applications are located in inland lakes and rivers, e.g., aquaculture and water quality monitoring. As mentioned earlier, UWSN is a typical heterogeneous wireless network, for which we take care of the energy heterogeneity factor. Assume that the three-dimensional network can be mapped into a two-dimensional network. Let *n* nodes be randomly deployed in a static M×M shallow monitoring water area. All nodes can be regarded approximately in the same plane, and more characteristics are as follows:

(1) Initially, each node is equipped with the different energy over the interval of $\left[E_{0},\left(1+\lambda \right)E_{0}\right]$, where $E_{0}$ is the lower bound (namely unit energy), and λ is a constant without upper bound to determine the value of the maximal initial energy, which satisfies λ>0.
(2) The sink is located at the center of the monitoring area, which is the only one not restricted by resources, such as energy, memory and calculation ability. Moreover, the sink can directly communicate with the gateway (base station).
(3) Owing to the adoption of clustering mechanism, non-cluster-head nodes are permitted to communicate with the cluster-head through single or multiple hops, while they cannot directly send packets to the sink.
(4) Each node is anchored at the specified area with a buoy, which means the network is relatively stable.
(5) Assume that the surface and the bottom of the water are relatively smooth planes, where the influence of underwater transmission delay and success rate for each node are the same.

### 3.2. Energy Consumption Model

Due to the different media between the underwater environment and the air, the traditional energy consumption in TWSN could not be applied to UWSN. To ensure the reliability of the communication, it may be necessary to send data multiple times, which accounts for a larger proportion of the entire energy consumption because of the additional propagation loss. Thus, the energy expended to transmit the *l*-bits message ($E_{tx}$) and to receive this message ($E_{rx}$) are, respectively, [21]:

(1) $$E_{tx}=l\cdot E_{elec}+\frac{l}{R}\cdot P_{t}$$

(2) $$E_{rx}=l\cdot E_{elec}$$

where $E_{elec}$ is the electronics energy dissipated per bit, *R* denotes the transmission rate (bit/s), and $P_{t}$ is the transmitted power. Specially, l/R expresses the time for sending the message.

Generally, there are two main acoustic signal propagation mechanisms (namely, Urick Propagation Model [22]) as the geometrical effect. One is cylindrical spreading, and the other is spherical spreading. Cylindrical spreading refers to sound propagation in the shallow water (i.e., depth lower than 100 m) with a cylinder bounded by the surface and the water bottom, while spherical spreading is for sound propagation between the sender and the receiver in the deep water (i.e., deeper ocean). According to the assumed network model, we focus on the cylindrical spreading for shallow water in this paper. Let $I_{t}$ be the current intensity, *A* be the cylindrical flank area, and then the transmitted power can be:

(3) $$P_{t}=A\cdot I_{t}=2\pi r\cdot H\cdot I_{t}$$

where *r* and *H* are the radius and the height of the cylinder, which refers to the distance between acoustic source and receiver as well as water depth, respectively.

To obtain $I_{t}$, the average intensity *I*, which is a plane wave with the root-mean-squared pressure *p* in a medium of density ρ and sound speed *c*, should be known previously, where $I=p^{2}/\rho c$ [23], and ρc is the acoustic impedance. Let SL denote the source level, the original $I_{t}$ can be expressed as the product of the intensity of the source level and the average intensity:

(4) $$I_{t}=10^{SL/10}\cdot \frac{p^{2}}{\rho c}$$

Specially, ρc is $1.5\times 10^{6}$ kg/(m${}^{2}$s) in some underwater environments. Thus, a plane wave of root-mean-squared pressure ($10^{-6}$ Pa) has an intensity of $0.67\times 10^{-18}$ W/m${}^{2}$ [23,24], and from Equation (4), we can get SL within logarithmic mode:

(5) $$SL=10\log\left(\frac{I_{t}}{0.67\times 10^{-18}}\right)$$

On the other hand, the source level can also be written by the passive sonar equation [21]:

(6) SL=SNR+TL+NL−DI

where *SNR* refers to the signal to noise ratio, and *TL* and *NL* are the transmission loss under the underwater environment and the noise level (i.e., ambient noise caused by turbulence, shipping, waves and thermal noise), respectively, *DI* denotes the directivity index and is 0 while using the omni-directional hydrophones.

Basically, the transmission loss (*TL*), which can be defined as the accumulated decreasing in acoustic intensity when an acoustic wave propagates outwards from the source [21], is a significantly important effect on sound communication in underwater. It can always be estimated by a variety of phenomena in underwater, e.g., geometrical spreading, absorption and scattering. With cylindrical spreading in shallow water, *TL* can be approximated as follows [24]:

(7) $$TL=10\log\left(r\right)+\alpha \left(f\right)\cdot r\times 10^{-3}$$

where α(f) refers to the absorption loss in medium using Thorpe’s equation [23]. According to Equations (3)–(7), the transmitted power ($P_{t}$) can be written as:

(8) $$P_{t}=C_{h}\cdot H\cdot r\cdot e^{\tilde{\alpha }\left(f\right)\cdot r}$$

where

(9) $$C_{h}=2\pi \times 0.67\times 10^{0.1(SNR+NL)-18}$$

(10) $$\tilde{\alpha }\left(f\right)=\alpha \left(f\right)\times 10^{-4}\times ln10$$

Finally, substituting Equation (8) into Equation (1), the energy consumption for sending a message is:

(11) $$E_{tx}=l\cdot E_{elec}+\frac{l}{R}\cdot C_{h}\cdot H\cdot r\cdot e^{\tilde{\alpha }\left(f\right)\cdot r}$$

## 4. The Proposed Algorithm-TCEB

From aforementioned energy consumption analysis, we can see that energy dissipated in underwater is quite different from the typical TWSN (energy consumption in TWSN can be found in [7]). Usually, the underwater acoustic communication needs to spend more resources without any energy recharge. Due to the non-uniform distribution of nodes, a node in the critical location (e.g., as the relay node in the communication link) sends the collected information, is prone to burden forwarding too much data. That will lead to network failure caused by premature node energy depletion. To be associated with the links situation, we use Markov model as the channel error model [25] in this paper. In addition, once the network is load imbalance, some nodes need to compete for communication channels queuing to send data, which greatly increases the end-to-end delay. Therefore, a topology control algorithm with energy balance (TCEB) using clustering technology for UWSN is proposed to consider how to improve energy efficiency and load balance to prolong the network lifetime as much as possible.

### 4.1. Non-Cooperative-Game-Based Cluster-Head Selection

With clustering technology, the number of cluster-heads in the network is critical to impact the final performance. To obtain the reasonable number of clusters and select nodes with more residual energy as the cluster-head is the goal of extending lifetime under the condition of limited resources. As far as we know, how to divide the entire network into clusters, i.e., how to determine the number of clusters, is a typical NP-hard problem [7]. Therefore, we use *N*th-order nearest-neighbor analysis theory [26] to adaptively calculate and obtain the optimal number of clusters (namely, $k_{opt}$). Moreover, the non-cluster-head node is only permitted to communicate with the sink through its cluster-head, which means the cluster-head should consume more energy on its extra communication and the collected data fusion. To ensure the load balance and the greatest degree of energy saving, the nodes in each cluster should be elected to be the cluster-head in turn. Thus, the non-cooperative game theory-based strategy in economics is adopted to determine which nodes are more suitable to be the cluster-head in each round of communication process. Notice that we only use the main idea from the non-cooperative game to make the energy balance.

In the non-cooperative game, each node can be selfish but rational, and then a node whether to being the cluster-head is depending on the game. Let G={N,S,U} be the game model, where *N*, *S*, and *U* are defined, respectively, as:

(1) $N=\{n_{1},n_{2},\ldots ,n_{n}\}$: Set of players, i.e., $n_{i}\in N$ is corresponding to node *i* in the network.
(2) $S=\{s_{1},s_{2},\ldots ,s_{n}\}$: Set of strategies, i.e., $s_{i}\in S$ ($s_{i}=0$ or 1) is a strategy of player *i* (i.e., node *i*), where $s_{i}=1$ refers to be the cluster-head, otherwise $s_{i}=0$, which means do not want to be the cluster-head.
(3) $U=\{u_{1},u_{2},\ldots ,u_{n}\}$: Set of payoffs, where $u_{i}\in U$ is the payoff function of player *i* (i.e., node *i*) if the node *i* is elected as the cluster-head.

To guarantee energy consumption balance, we consider node’s energy and path loss as the main factors of cluster-head selection. Thus, the payoff function $u_{i}$ can be further expressed as:

(12) ui=βEr(i)E¯r+(1−β)1∑j∈Neiipl(i,j)∑j∈Neiipl(i,j)qiqi

where $E_{r}\left(i\right)$ and ${\bar{E}}_{r}$ are the residual energy of node *i* and the average residual energy of the whole network in current round, respectively; pl(i,j) denotes the path loss of node *i* to its one-hop neighbor node *j*; $Nei_{i}$ and $q_{i}$, respectively, refer to the set of one-hop neighbor nodes and its quantity of the node *i*; and β is a constant adjustment factor which satisfies 0<β<1. Notice that ∑j∈Neiipl(i,j)∑j∈Neiipl(i,j)qiqi denotes the average path loss of node *i* to its one-hop neighbor nodes.

According to the non-cooperative game theory, the player chooses the optimal strategy from the set of strategies to obtain the greater profit. From Equation (12), we can find that the higher the residual energy of the node and the lower the average path loss of the node to its one-hop neighbor nodes, the greater the payoff of the node has. To balance the energy consumption of the network, we make a rule that the node which is being a cluster-head can get better profit. In that case, we move to see which one has obtained the greater profit. In other words, the node which has greater $u_{i}$ is easier elected with high probability to be the cluster-head. In addition, β is used for adjusting the proportion of the energy and path loss so that we can obtain an optimum payoff for each node, i.e., this is a trade-off between the energy and the path loss.

The detailed steps for cluster-head selection can be described as follows. Algorithm 1 shows the pseudo-code of the cluster-head selection.

- **Step 1** A triad game model, namely, G={N,S,U} is firstly built for the network.
- **Step 2** According to current node distribution, the optimal number of clusters (i.e., $k_{opt}$) is calculated through *N*th-order nearest-neighbor analysis theory [26].
- **Step 3** Each node broadcasts the *HELLO* message with its maximum transmission power and set a *timeout* for waiting for the reply message. Meanwhile, it collects the neighbor node’s message under the specified *timeout* and establishes its neighbor list.
- **Step 4** Each node calculates its own payoff (i.e., $u_{i}$) according to Equation (12), and then broadcasts its calculation result of $u_{i}$ within the *timeout*.
- **Step 5** A node receives the payoff ($u_{i}$) of its neighbor node and stores $u_{i}$ to the corresponding node in the neighbor list. After getting all payoffs of nodes in the neighbor list, each node sorts the payoffs (includes itself) out in terms of descending order.
- **Step 6** According to the optimal cluster number, the former $k_{opt}$ nodes with greater $u_{i}$ are selected as the final cluster-head by the game from the network. Then, the newly elected cluster-heads broadcast the message that they have been selected as the cluster-head of the $k_{opt}$ cluster. That means all $k_{opt}$ nodes make a decision to be cluster-head in this round during the game, and the process of cluster-head selection is ended.

**Algorithm 1** Cluster-Head Selection**Require:** A triad game model G=(N,S,U)
1:Initialize the network where n[i]← ‘N’2:Calculate $k_{opt}$ using *N*th-order nearest-neighbor analysis method from [26]3:$n_{i}$ broadcasts the *HELLO* and set a *timeout*4:**while**t<timeout**do**5:    $n_{i}$ collects its neighbor node’s *HELLO*6:**end while**7:$n_{i}$ establishes its neighbor list8:$n_{i}$ calculates its $u_{i}$ according to Equation (12)9:$n_{i}$ broadcasts its $u_{i}$ and set a *timeout*10:**while**t<timeout**do**11:   $n_{i}$ receives payoff ($u_{i}$) of its neighbor nodes and stores in the neighbor list12:**end while**13:$n_{i}$ sorts the payoffs ($u_{i}$) out by descending order14:chs← Chooses the former $k_{opt}$ nodes with greater $u_{i}$ as the cluster-head15:**for**k←1**to**$k_{opt}$**do**16:    n[chs[k]]← “C”17:    $n_{chs[k]}$ broadcasts its message of newly elected to be cluster-head18:**end for**


Through the process of cluster-head selection, we can find that it is precisely because of the introduction of the game model and node’s payoff function. The $k_{opt}$ cluster-heads are correctly elected under the premise of the comprehensive balance of energy consumption and path loss. Then, the intra-cluster and inter-cluster topology are beginning to construct one after the other.

### 4.2. Intra-Cluster Topology Construction

After the non-cooperative-game-based cluster-head selection, the cluster-heads have been exactly determined as well as the probably partitioned region of the cluster. However, the current clusters constitute only the basic topology so that communication on intra-cluster and inter-cluster is further to build to carry out the data transmission. Usually, the underwater acoustic communication is affected by multipath and high end-to-end delay, while also depending on the distance and the time as well as the frequency. Therefore, topology construction will have a great impact on network communication performance.

**Figure 1:**
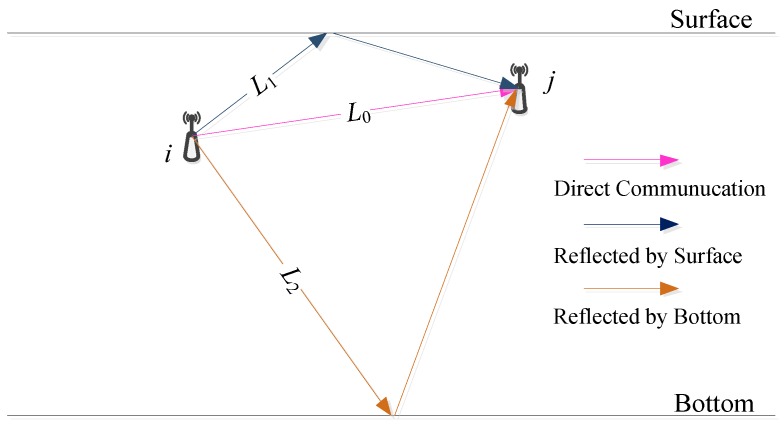
Multipath propagation in shallow water.

Initially, we take the delay with the multipath effects into consideration when the intra-cluster topology is built. Due to the non-uniformity of the underwater medium space, the acoustic channel has a multipath phenomenon. That means the signal from the source to the destination may pass through the different path under a certain transmission power. On the other hand, because of the difference of path length, the acoustic wave which is to reach the destination by different paths takes a different time, as well as signal attenuation. As shown in Figure 1, multiple paths exist from the source node *i* to the destination node *j*. They can roughly be represented by $L_{0}$, $L_{1}$, and $L_{2}$, respectively, where $L_{0}$ indicates the direct path between the source and the destination, $L_{1}$ denotes that a reflection arrives through the water surface, and $L_{2}$ is a reflection as well but through the water bottom. Obviously, $L_{0}$ is the shortest path for acoustic wave propagation, which costs the minimum duration with the less delay.

The delay is complicated to correctly calculate in the practical underwater environment, thus we show the scenario in Figure 1. Inspired by Ibrahim et al. [27], the total delay between the node *i* to *j* can be written as:

(13) $$delay_{i\to j}=\frac{l}{C}+\frac{d\left(i,j\right)}{c}+\Delta \tau$$

where *l* and *c* are the length of the data packet in bits and the propagation velocity of the acoustic wave in water, respectively. d(i,j) refers to the distance between the node *i* and *j*, Δτ is the delay caused by multipath propagation. *C* is the channel capacity in bits per second which can be expressed according to the Shannon’s theorem:

(14) $$C=B\cdot \log_{2}\left(1+SNR\right)$$

where *B* is the bandwidth of the channel, and SNR refers to the signal to noise ratio. Assuming that the noise is Gaussian and the channel is time-invariant for some interval time, the capacity can divide the total bandwidth into many narrow sub-bands [28]. The *i*th sub-band is centered around the frequency $f_{i}$ (i=1,2,…), and its width is Δf. We introduce the power spectral density of the signal while considering the real scenario. Therefore, more general channel capacity [29] can be expressed as:

(15) $$C=\sum_{i}\Delta f\log_{2}^{\left[1+\frac{X(f)H(f)}{N(f)}\right]}$$

where X(f) is the power spectral density of the transmitted signal from the source, H(f) denotes the channel transmission function, and N(f) is the noise power spectral density.

Let $delay_{i\to j}^{0}=\frac{l}{C}+\frac{d_{0}\left(i,j\right)}{c}+\tau_{0}$ denote the total delay when the signal is propagating on path $L_{0}$. To reduce the complexity of delay analysis by multipath effects, the communication paths from the source to the destination would be restricted in this paper, which means data propagation through the reflection path is limited. Therefore, according to this rule, the path can be defined invalid if the delay for signal propagation from the node *i* to *j* on such path (any path except for $L_{0}$) is greater than $delay_{i\to j}^{0}$.

When the intra-cluster topology construction is beginning, all the non-cluster-head nodes wait to join one of the $k_{opt}$ clusters. After broadcasting the *INVITATION* message including the ID and $u_{i}$ by the cluster-head, the non-cluster-head node will perform the next action promptly depending on how many *INVITATION* messages it has gotten. Considering the case that the non-cluster-head node may have received a number of *INVITATION* messages from the multiple cluster-heads, various strategies are adopted according to the value of $u_{i}$ to make all of the clusters load balance as much as possible. Once the non-cluster-head node receives only one *INVITATION* message, it naturally becomes the cluster member of the cluster-head which sends such message previously and replies the *ACK* message. If the non-cluster-head node receives more than one *INVITATION* message, then it chooses to be the cluster member of the cluster-head with the largest $u_{i}$ and sends back the reply message of *ACK*. Otherwise, the non-cluster-head node waits to join a cluster with multi-hop communication method. Specially, once the non-cluster-head node receives multiple *INVITATION* messages from different cluster-heads with the same largest $u_{i}$, then it chooses one of the clusters randomly to join while answering the corresponding *ACK* back.

During such process, we notice that non-cluster-heads may not have received any messages from any cluster-head. To achieve the goal of minimizing the energy consumption of the network, it is necessary to design a strategy on how to select the next hop neighbor node to form the best transmission path. Consequently, the relay node will be selected in terms of the communication cost to make sure that kind of node joins into one of the clusters. The mechanism for selecting the relay node can be defined as:

(16) $$\begin{array}{c} p\left(i,j\right)=\gamma \frac{E_{r}\left(j\right)}{E_{\mathrm{cons}}\left(i,j\right)}+\left(1-\gamma \right)\left(R_{\mathrm{link}}\left(i,j\right)+\frac{1}{pd_{\mathrm{loss}}\left(j\right)}\right) \end{array}$$

where $E_{r}\left(j\right)$ is the residual of the node *j*, $E_{cons}\left(i,j\right)$ is the total energy consumption on communication between the node *i* and *j*, which refers to both energy dissipation when the node *i* sends the message and the node *j* receives the message. $R_{link}\left(i,j\right)$ and $pd_{loss}\left(j\right)$ are the link reliability between the node *i* and *j* and the packet loss rate of the node *j*, respectively, and γ is the adjustment factor, which satisfies 0<γ<1.

Equation (16) reflects the probability that the non-cluster-head node *i* chooses its one-hop neighbor node *j* as the relay node to communicate with the cluster-head. It can be easily seen from Equation (16) that the more the residual energy of the node *j* and the lower the cost between both communication nodes, the easier the node *j* to be selected as the relay node. Simultaneously, the higher the link reliability and the smaller the packet loss rate, the easier the node *j* to be the relay node as well. Therefore, to obtain an optimum relay node during the intra-cluster topology construction, we cannot merely look at a parameter or a part of the parameters in this strategy, but take care the trade-off between the various parameters. At this time, γ is the key factor for adjusting the trade-off to select the optimum relay nodes to build better local topology.

Algorithm 2 gives the pseudo-code of intra-cluster topology construction. The detailed steps for intra-cluster topology construction are presented as follows.

- **Step 1.** The cluster-head broadcasts the *INVITATION* message including its ID and $u_{i}$, and set a *timeout* as well.
- **Step 2.** Once the non-cluster-head can receive the *INVITATION* message from the cluster-head within *timeout*, go to **Step 3**, otherwise, perform **Step 5**.
- **Step 3.** Once the non-cluster-head node receives various *INVITATION* messages from multiple cluster-heads, it will choose to join the cluster which has the cluster-head with the largest $u_{i}$. If there is existing an equally largest $u_{i}$ from different cluster-heads, then go to **Step 4**, otherwise, reply an *ACK* message and perform **Step 6** directly.
- **Step 4.** The non-cluster-head randomly chooses one of the clusters which have the same largest $u_{i}$, and then it responses the *ACK* reply.
- **Step 5.** Once the non-cluster-head does not receive any *INVITATION* message from any cluster-head, it has to select the relay node according to Equation (16). Afterwards, the non-cluster-head will establish the communication link with the relay node.
- **Step 6.** The intra-cluster topology keeps going on constructing until all non-cluster-heads join one of the $k_{opt}$ clusters.

**Algorithm 2** Intra-cluster topology construction
1:
**for** k←1 **to** $k_{opt}$ **do**
2:    $chs_{k}$ broadcasts the *INVITATION* and set a *timeout*3:
**end for**
4:
**for** i←1 **to** *n* **do**
5:    **if** n[i]!= ‘C’ **then**6:        **if** $n_{i}$ receives the *INVITATION* within *timeout* **then**7:            **if** Num(INVITATION)>1 **then**8:                **if** there is no equal $u_{i}$ **then**9:                    $n_{i}$ becomes the cluster member of the cluster-head with the largest $u_{i}$10:               **else**11:                  $n_{i}$ randomly chooses one of clusters to join12:               **end if**13:           **else**14:               $n_{i}$ becomes the cluster member of the cluster-head which sends *INVITATION*15:           **end if**
16:           $n_{i}$ replies an *ACK*17:       **else**18:           $n_{i}$ selects the relay node *j* according to Equation (16)19:           comm[i,j]←120:       **end if**21:    **end if**
22:
**end for**



### 4.3. Inter-Cluster Topology Construction

The inter-cluster topology construction is the final process to connect all of the parts (i.e., clusters) as a tree for further communication requirement. It actually is how to build the communication link between the clusters and the sink. That is the construction of the global network if the intra-cluster topology construction can be regarded as the local network formation. Consequently, in this section, we need to find a way to rapidly build the path from the cluster-head to the sink. Specially, the cluster-head which is far away from the sink should be give more care, because it cannot directly communicate with the sink by one-hop transmission. It must rely on another cluster-head that is closer to the sink by multiple-hop communication to complete the task of data transmission.

**Figure 2:**
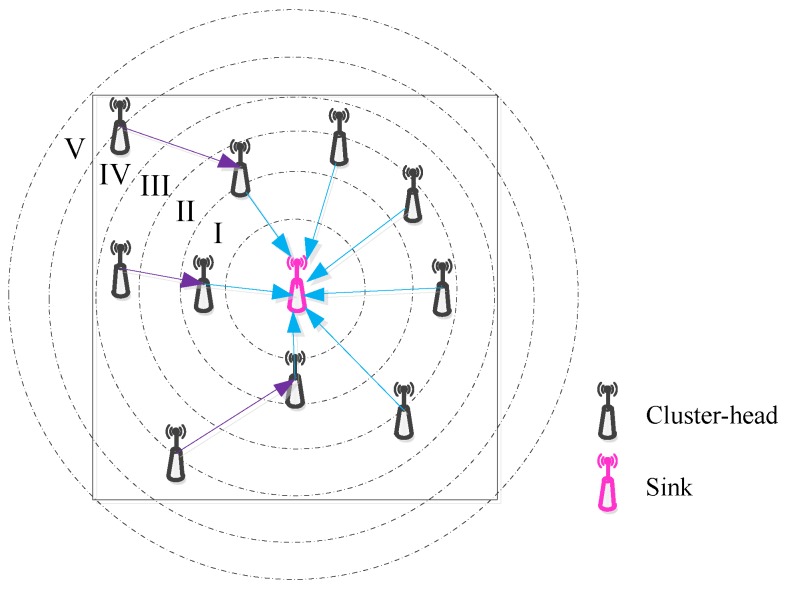
Divide the hierarchical area with the broadcast radius of the sink.

When the inter-cluster topology is tried to build, it begins from the sink. Initially, the sink broadcasts the *HELLO* message to the monitoring area as its minimum transmission power, and then gradually increases until its maximum transmission power or the communication radius can reach over the entire monitoring region. As shown in Figure 2, according to the power level of the sink, it can be recorded as level I, level II, level III, etc. (from low to high), which are corresponding to the different ring monitoring area. If a cluster-head receives the *HELLO* message within one of the broadcast ranges corresponding to a power level of the sink, then it records its current power level.

Once the grading is completed, the cluster-head with the lowest power level of the sink (e.g., the area in which the power level I is located in Figure 2) first starts to establish the communication relationship with the sink. Then, the cluster-head within power level II is considered to be next task that makes the connection to the sink. Whether it chooses one of the cluster-heads within power level I as the relay node or still keeps directly communicating with the sink depends on the communication cost it spends. The main cost function of communication between the cluster-head *i* and the cluster-head *j* (may be the sink) is given as:

(17) $$\cos t\left(i,j\right)=\omega \frac{E_{r}\left(i\right)}{E_{Init}\left(i\right)}+\left(1-\omega \right)\frac{d\left(i,j\right)}{r_{sens}\left(i\right)}$$

where $E_{r}\left(i\right)$ and $E_{Init}\left(i\right)$ are the residual energy and the initial energy of cluster-head *i*, respectively; d(i,j) refers to the distance between cluster-head *i* and *j*; and $r_{sens}\left(i\right)$ is the sensing radius of the cluster-head *i*. ω denotes the adjustment factor, which can adjust the proportion relationship of the residual energy and the communication distance.

**Algorithm 3** Inter-cluster topology construction
1:Initialize power level of $k_{opt}$ cluster-heads power[k]←02:**for**i←1**to** maxPower(*sink*) **do**3:    **if** power[k]==0 && $chs_{k}$ receives *HELLO* from the sink **then**4:        power[k]←i5:    **end if**6:
**end for**
7:
k←1
8:
**while** $k\leq k_{opt}$ **do**
9:    **if** power[k]>1 **then**10:      best←cost(k,sink) // Initial best cost by Equation (17)11:      relay←sink12:      **for** j←1 **to** $k_{opt}$ **do**13:          **if** cost(k,j)+cost(j,sink)<best **then**14:              best←cost(k,j)+cost(j,sink)15:              relay←j16:          **end if**17:      **end for**18:      comm[chs[k],chs[relay]]←119:   **else**20:      comm[chs[k],sink]←121:   **end if**22:   k←k+123:
**end while**



Through Equation (17), we can make a correct decision whether the current cluster-head needs the relay cluster-head to forward the data to the sink. If the cost of the cluster-head spent is indeed less than previous communication way (i.e., directly communicate with the sink) via the relay cluster-head, then it establishes the multi-hop communication between the cluster-head and the sink. In that way, it also enhances the close connection between the different inter-clusters simultaneously. Traversing all of the cluster-heads of each power level one after another, the process of inter-cluster topology control will be continued until all of the cluster-heads have already built the direct or indirect connection with the sink. Algorithm 3 presents the pseudo-code of the detailed inter-cluster topology construction.

Afterwards, the collected data can be prepared to forward through the intra-clusters and the inter-clusters. Both cluster-head and non-cluster-head will perform the communication task according to their TDMA scheduling table. On the other hand, during the process of the intra-cluster and the inter-cluster topology construction in every round, each non-cluster-head or cluster-head also stores its corresponding routing information to prevent failures of the node and the link in TCEB algorithm. That could make a quick response and restart the topology reconstruction, i.e., the process of topology maintenance is triggered to perform immediately.

## 5. Simulation

### 5.1. Simulation Environment

To validate the effectiveness of the proposed algorithm, we implement and evaluate TCEB with the typical clustering-based algorithms LEACH [7], EDCS [8], and GR-CTC [10] under almost the same parameters setting in Matlab R2013a and Atarraya [30]. As we discussed before, all nodes are randomly deployed in a square of 100 m × 100 m, and the sink is located at the center of the area, i.e., (50, 50). More detailed parameters which we used in the simulation are given in Table 1. Particularly, the weighted factors, such as β, γ, and ω, are taken from the results of hundreds of experiments.

During the simulation, we mainly make two kinds of testing experiments. One is for self-comparison which is to observe the efficiency of TCEB under different node numbers and energy factors. The other is for comparison with typical clustering-based algorithms to find whether TCEB has preferable merits. To eliminate the randomness of the experimental results, all of the tests are performed at least 100 times to get the average value.

### 5.2. Partial Parameters Impact Analysis

In this section, we firstly focus on the energy-efficiency of TCEB, i.e., the changes of network lifetime under the different number of nodes and energy heterogeneity factor in UWSN. Usually, the lifetime contains two periods: the stable phase and the unstable phase. The stable phase refers to the time when the first node dies, and the unstable phase denotes the period between when the first node dies and the last node dies. The round is presented as the unit of the network lifetime. Consequently, the variational trend of both periods should be watched out when the number of nodes and energy heterogeneity parameter change.

**Figure 3:**
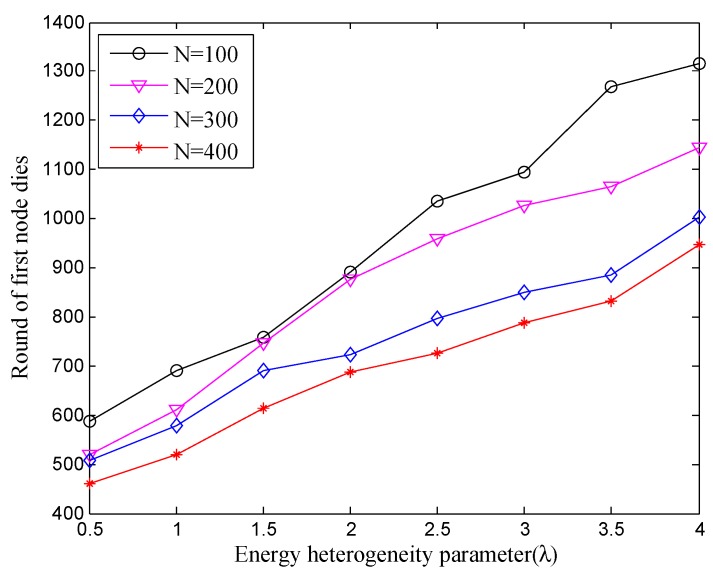
The stable period with different number of nodes under different energy heterogeneity factor.

**Figure 4:**
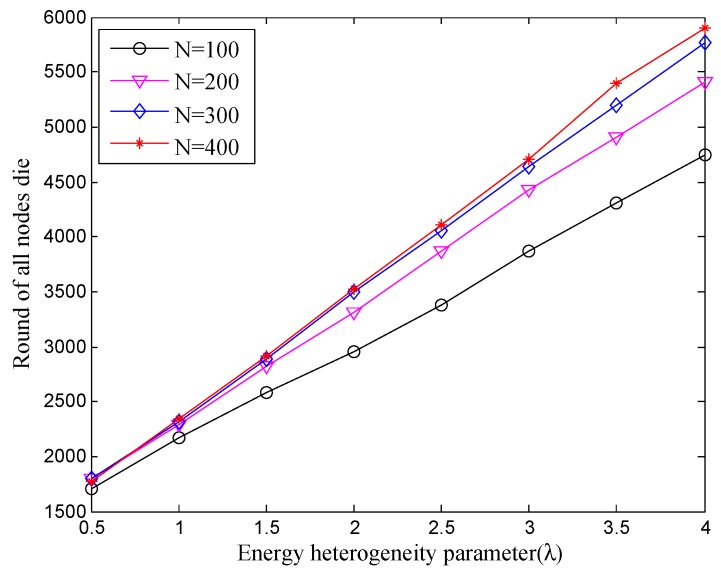
The whole lifetime with different number of nodes under different energy heterogeneity factor.

Figure 3 and Figure 4 show the variation of the stable period and the whole lifetime with the different number of nodes under different energy heterogeneity factor, respectively. In Figure 3, with the increase of λ, i.e., as nodes are deployed with more energy, the dead time of the first node will become later than before, no matter what the current number of nodes is, vice versa. Simultaneously, from the longitudinal observation, as more nodes are deployed, the first node dies earlier under the same energy heterogeneity factor (λ). This is because the number of exchange messages between nodes and the interference in the network will sharply increase when the number of nodes is greater than a certain value. Obviously, it may cause dissipating more additional energy during the networking, which directly leads to the first node dead earlier. Therefore, more nodes is not always better, especially in a certain specific concentration area.

As shown in Figure 4, with the increase of λ, the lifetime also becomes longer under the different number of deployed nodes. Generally, increasing the number of deployed nodes is equivalent to increasing the energy of the network. Nevertheless, we can find that the whole network lifetime does not vary greatly under the same λ while increasing the number of deployed nodes. Similar to the situation of the first node dead, the main reason is located at the increased frequency of communication and the occurrence of interference. It spends extra energy on exchanging the messages and controlling the topology, while increasing the total energy of the network. Consequently, enlarging the energy heterogeneity factor can definitely extend the network lifetime, while the number of nodes should be deployed in reasonable quantity in the practical underwater scenarios.

**Figure 5:**
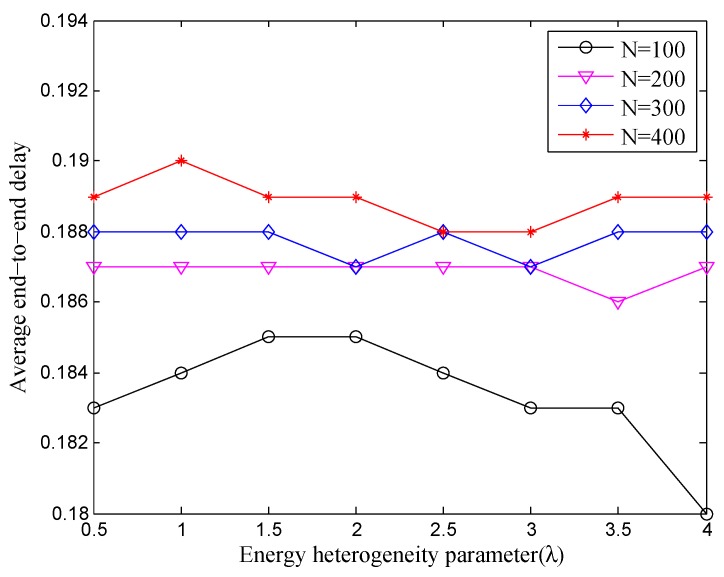
The average end-to-end delay (s) under different energy heterogeneity factor.

We also pay close attention to the impact of the end-to-end delay caused by the change of nodes number and energy heterogeneity parameter in UWSN. The variation of the average end-to-end delay which is under the different *n* and λ in TCEB is shown in Figure 5. As the energy heterogeneity factor λ enlarges, basically, the average end-to-end delay does not change much under the scenario of the same deployed nodes. The changes are only milliseconds, which means equipping more energy almost doe not influence the end-to-end delay. At the same time, it is also reflected from the side that the topology is timely scheduled to make nodes adapt to the new roles by dynamic adjustment, which ensures the QoS of the network. Furthermore, with the increase in the number of deployed nodes, the average end-to-end delay inevitably rises. This is because the interference among nodes also gradually increases when the number of deployed nodes has reached a certain degree of the saturation. Apparently, it could inadvertently reduce the QoS of the network. Consequently, we come to the conclusion again from the average end-to-end delay that more nodes is not always better in the network, even though many nodes can provide much more total energy.

**Figure 6:**
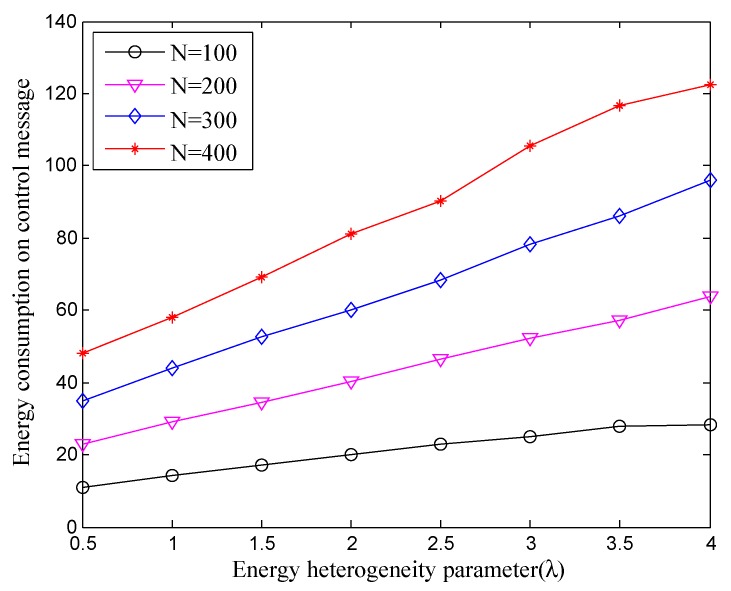
The total energy consumption (J) for control message of the whole network under different energy heterogeneity factor.

In addition, we also need to investigate the overhead on control message since it is related to the lifetime and final energy consumption of the entire network. As shown in Figure 6, we can see the total energy consumption on control message is rising for any network scale (i.e., n=100, 200, 300, 400) with increasing energy heterogeneity parameter λ. This is because the network which has more energy should have a longer lifetime at work, and the energy overhead on control message will continue. Meanwhile, from the longitudinal observation, enlargement of network scale not only provides more total energy of the network, but also increases overhead on control message. Obviously, it is a normal phenomenon that it brings the additional communication between nodes when more nodes are added into the network. Fortunately, it has not grown exponentially and is still within acceptable limits. Finally, the overhead on the control message accounts for 10–20% of the total communication cost by the statistic.

### 5.3. Performance Evaluation

In this section, we compare the performance of TCEB with typical LEACH, EDCS, and GR-CTC in the underwater environment. We pay attention to when the first node and all of the nodes are dead, the average end-to-end delay, and the throughput of the network. For the first two aspects, we focus on the energy-efficiency which is presented as the time period (namely, round) including the stable phase and the whole lifetime of the network. Moreover, the latter is to examine the communication performance of the network. It also reflects from the side the QoS, e.g., the packet delivery loss rate, which is too high to result in the substantial increase of the last received data packets. All of the algorithms are evaluated under different deployed nodes density (n=100 or n=200) with equipping different energy, where the results are averaged over multiple times to eliminate the randomness.

**Figure 7:**
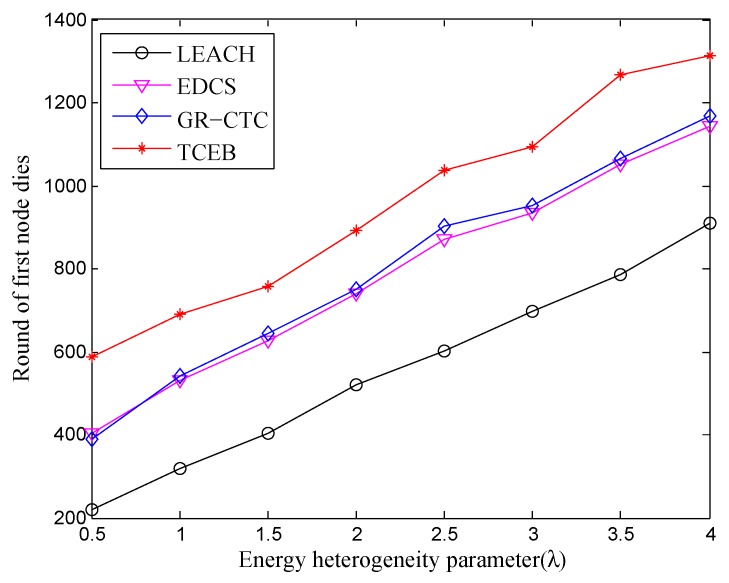
The comparison of stable period under different energy heterogeneity factor (n=100).

**Figure 8:**
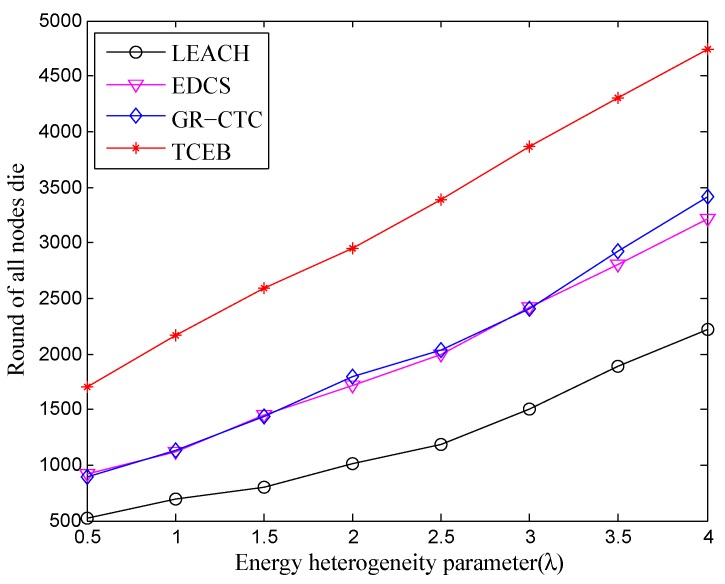
The comparison of whole lifetime under different energy heterogeneity factor (n=100).

Figure 7 and Figure 8 are, respectively, given to show the comparison of four algorithms into the stable phase (i.e., the time when first node is dead) and the whole lifetime (i.e., the time when all nodes are dead) under different energy heterogeneity factor, in which the number of deployed nodes is 100. Basically, with the enlargement of λ, the death of the first node and the death of all nodes are postponed in four algorithms. That means to equip more energy for each node can help prolong both the stable period and the whole lifetime. Nevertheless, from a longitudinal comparison, the TCEB is better than the three other algorithms under the same λ in both stable period and whole lifetime. Obviously, the typical LEACH has the worst energy-efficiency since it has a shorter lifetime. As far as we know, LEACH was once the classical clustering-based algorithm for the homogeneous TWSN. However underwater is a very complicated environment, and easily cut LEACH down on communication efficiency due to various factors. Since LEACH cannot respond to a variety of underwater emergencies, it would not be suitable for applying to UWSN which is a special heterogeneous wireless network.

On the other hand, EDCS and GR-CTC are also better than LEACH in the lifetime under the network scale of 100 nodes, where the EDCS is designed for general heterogeneous wireless sensor network and the GR-CTC is proposed for UWSN. In EDCS, many impact factors or existing difficult problems have been concerned in design, but it still ignores the complex status of underwater acoustic communication. The same packets may be forwarded multiple times since there might be higher packet delivery loss rate. That is the reason to lead to dissipating more energy on resending the packets. We can find there is a centralized strategy adopted in GR-CTC which makes the extra interaction between the nodes. It spends some energy and reduces the efficiency of the GR-CTC, even if it is specifically designed for underwater scenarios. Unlike with LEACH, EDCS, and GR-CTC, the proposed TCEBt combines the characteristics of the heterogeneous network and the particularities of the underwater environment; simultaneously, the larger underwater energy consumption and the multipath propagation, as well as the link stability, are all taken into account to guide to select the cluster-heads and build the multi-hop tree topology. Moreover, with a serial of strategies of load balance on energy consumption between nodes, the TCEB prolongs both the stable period and the whole of the lifetime, naturally.

**Figure 9:**
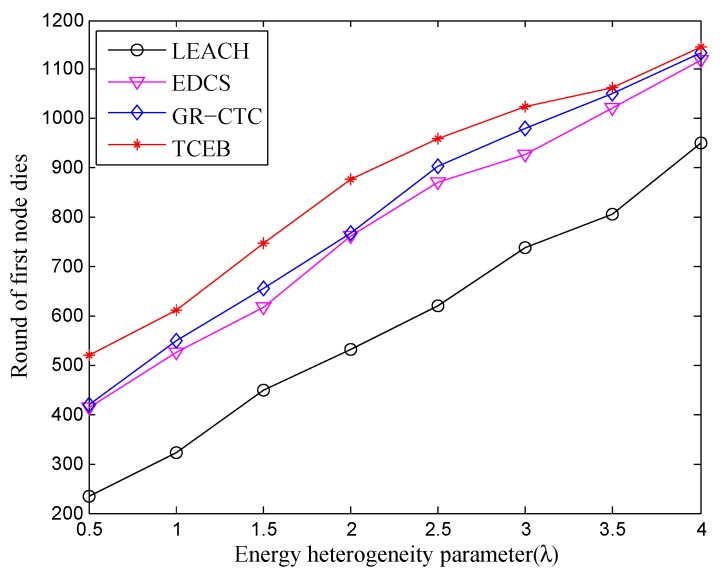
The comparison of stable period under different energy heterogeneity factor (n=200).

**Figure 10:**
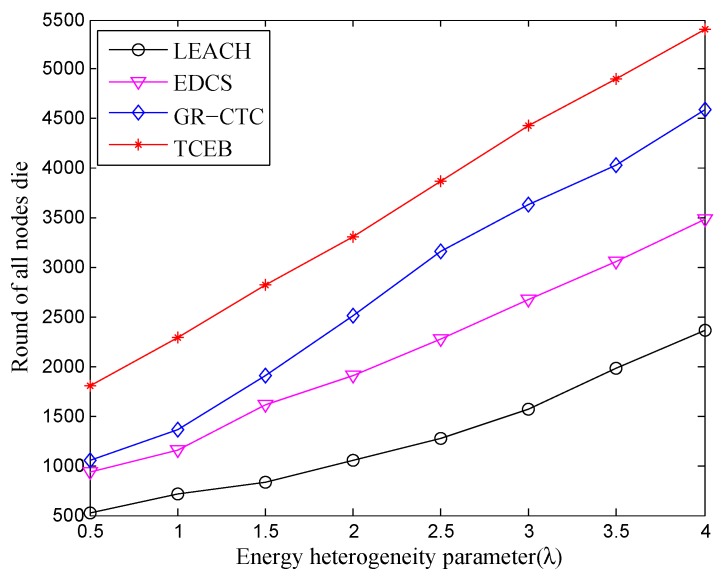
The comparison of whole lifetime under different energy heterogeneity factor (n=200).

Next, we observe the comparison of the stable period and the whole of lifetime under the nodes density of n=200, when the energy heterogeneity factor (λ) increases. As shown in Figure 9 and Figure 10, we can see almost the same trend of the curve while comparing with Figure 7 and Figure 8, respectively. That is, the stable period and the whole lifetime of the four algorithms are all extended with the increasing of λ under the network density of n=200. However, from the comparison of Figure 7 and Figure 9, we can find that the first node is dead relatively earlier under n=200 than the scenario of n=100 within the same energy heterogeneity factor in most of the algorithms. The main reason is there are too many nodes located in the same smaller monitoring area, which would exchange much more messages in the local network. At the same time, too many nodes inevitably make much more communication interference among clusters and nodes. It could result in nodes failure or even the partial network paralysis. Specially, the complex underwater environment may cause increasing the number of link-hops, which expands the burden of the relay nodes. Hence, it leads to depleting extra energy on frequently resending the data to enable the normal work and make the entire network load balance as well as stabilization.

In Figure 10, the lifetime in TCEB is evidently better than other three algorithms. Without loss of generality, combined with aforementioned analysis, the TCEB has the merit of energy-efficiency over the other three algorithms under different network density even if there are equipped with the different energy heterogeneity parameter. From the energy-efficiency point of view, the proposed TCEB can contribute to currently further underwater applications.

**Figure 11:**
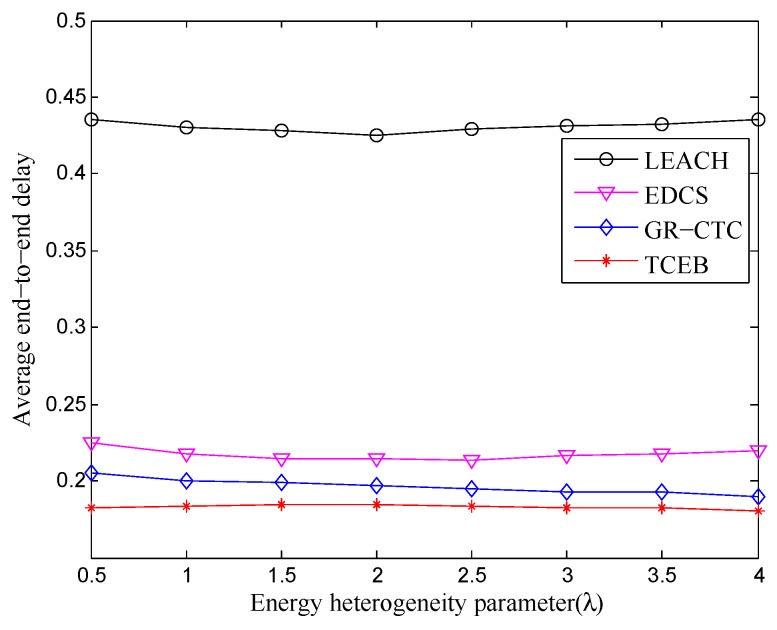
The comparison of average end-to-end delay under different energy heterogeneity factor (n=100).

**Figure 12:**
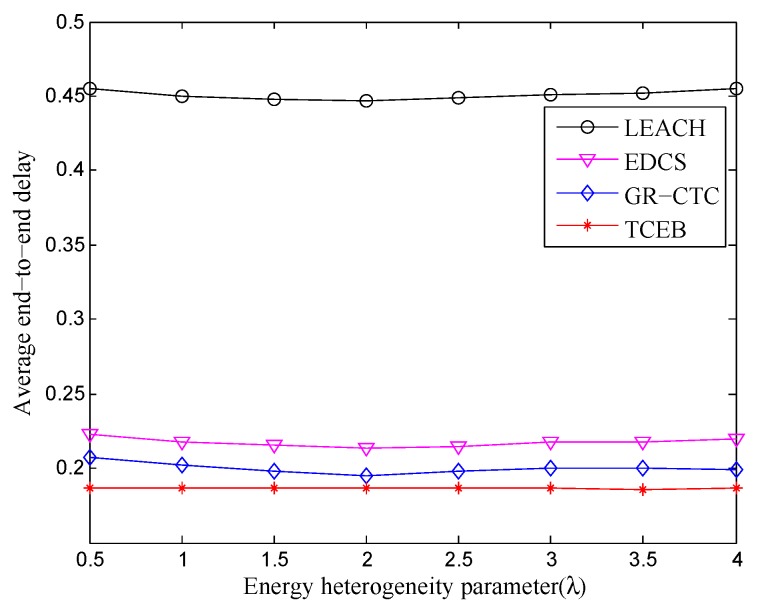
The comparison of average end-to-end delay under different energy heterogeneity factor (n=200).

Furthermore, Figure 11 and Figure 12 show the comparison of average end-to-end delay in TCEB and other typical algorithms under n=100 and n=200, respectively, when the energy heterogeneity factor (λ) changes. In Figure 11, we can clearly see that there is only tiny fluctuation for the curve of each algorithm under different λ. That means the average end-to-end delay is relatively stable under n=100 when the total energy of the network changes (i.e., λ changes). Meanwhile, compared with other algorithms, TCEB has less average end-to-end delay because it takes more real situations into consideration. In contrast, LEACH has highest average end-to-end delay within four algorithms since it is more suitable for ideal homogeneous environments. As shown in Figure 12, it reflects almost the same trends for each of algorithm under n=200 while comparing with the scenario of n=100. However, in Figure 11 and Figure 12, we find that the average end-to-end delay for each algorithm is rising when the network scale enlarges, but the varied amplitude is small. This is because each algorithm adopts more or fewer mechanisms to reduce the end-to-end delay.

**Figure 13:**
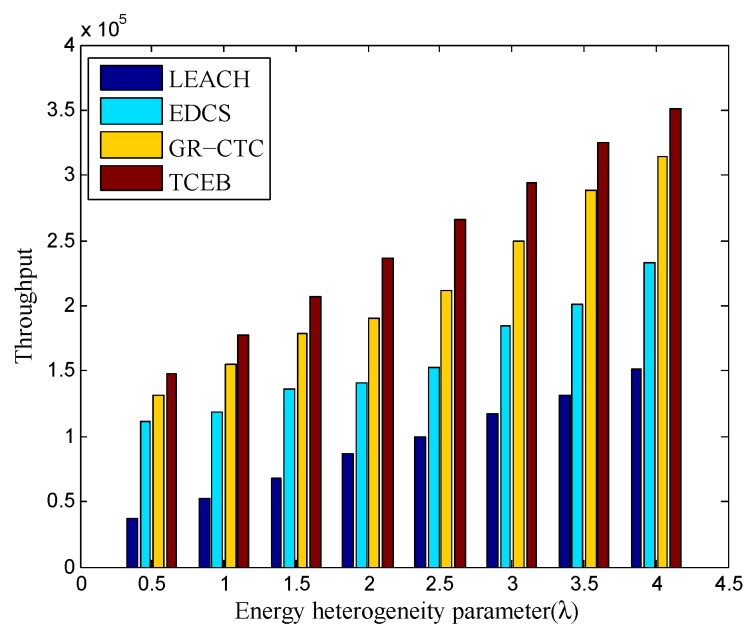
The comparison of throughput under different energy heterogeneity factor (n=100).

**Figure 14:**
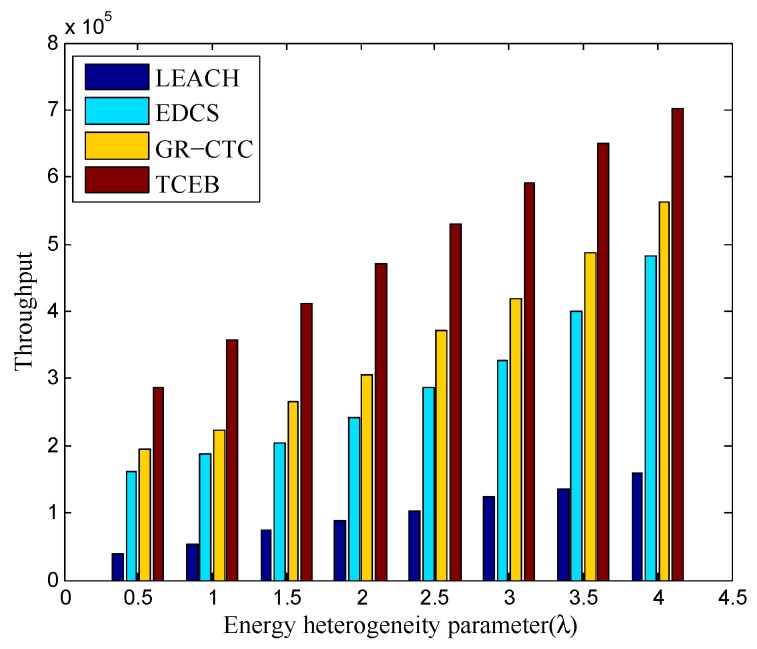
The comparison of throughput under different energy heterogeneity factor (n=200).

Finally, Figure 13 and Figure 14 show the comparison of throughput in TCEB and other typical algorithms under n=100 and n=200, respectively, when the energy heterogeneity factor (λ) changes. We can clearly see the throughput is increased in all four algorithms under different network density (both n=100 and n=200) when the λ becomes larger. Obviously, it follows the rule that the more energy the network equips, the higher the throughput is. On the other hand, no matter the network density is 100 or 200 nodes, the throughput in TCEB is higher than the three other algorithms with the same λ. This is because TCEB has more efficient clustering-based scheme with a load balance strategy to deal with the complicated underwater communication. It not only saves energy and prolongs the lifetime of the network, but also brings the higher throughput. In addition, it also reflects from the other side that the TCEB has higher transmission success rate than the three other algorithms, thereby increases the throughput of the network.

## 6. Conclusions

In this paper, we address the issue of energy-efficiency with longer time-delay and multipath effect in underwater wireless sensor networks and propose a topology control with energy balance scheme to ensure the network load balance and prolong the lifetime. Combined with the path loss and the energy as well as the average energy of current network, the proposed TCEB adopts the idea of game-based scheme to select the nodes with better payoff as the cluster-head. Both intra-cluster and the inter-cluster topology construction are built to choose the reasonable candidates of relay nodes to form the optimum links in the underwater network. With the help of topology maintenance, TCEB also has an ability to dynamically adjust the topology when the underwater network is unavailable or non-optimal. Simulation results show that TCEB is efficient to prolong the network lifetime, and its performance is better than LEACH, and outperforms EDCS and GR-CTC as well.

## Figures and Tables

**Table 1:** Network simulation parameters setup.

Parameters	Values
Network size (M×M)	(100 m × 100 m)
Location of sink	(50,50)
Node number (n)	100, 200, 300, 400
Energy heterogeneity factor (λ)	[0.5, 4.0]
Initial energy $\left(E_{0}\right)$	0.5 J
$E_{elec}$	50 nJ/bit
Depth (H)	10 m
Transmission rate (R)	2000 bit/s
β	0.8
γ	0.7
ω	0.5
